# STAT3 regulated *miR-216a* promotes ovarian cancer proliferation and cisplatin resistance

**DOI:** 10.1042/BSR20180547

**Published:** 2018-08-29

**Authors:** Pengfei Jin, Yanjun Liu, Ruijuan Wang

**Affiliations:** Department of Obstetrics and Gynecology, Liaocheng People’s Hospital, No.67 of Dongchang West Road, Liaocheng 252000, Shandong, China

**Keywords:** Cisplatin, Ovarian cancer, STAT3, miR-216a

## Abstract

Cisplatin is the first-line treatment for ovarian cancer. However, the clinical outcome of cisplatin treatment in ovarian cancer is hindered by cancer resistance. Here we aim to explore the role and mechanism of *miR-216a* in the cisplatin resistance of ovarian cancer. The effects of *miR-216a* overexpression and inhibition on ovarian cell proliferation, colony formation, and cisplatin resistance were investigated by MTT assay and soft agar colony formation assay. Bioinformatics analyses using TargetScan and rVista, qPCR, and luciferase assay were also used to explore and verify downstream effectors and regulators of *miR-216a*. Proliferation, colony formation, and cisplatin resistance of ovarian cancer cells are promoted by *miR-216a* overexpression but inhibited by *miR-216a* inhibition. PTEN is a direct target of miR-216a and PTEN expression antagonizes the tumor-promoting function of *miR-216a*. STAT3 is a regulator of *miR-216a*, and PTEN is also regulated by STAT3. *miR-216a* up-regulation is associated with cisplatin resistance in ovarian cancer and this effect is mediated by PTEN. STAT3 is a regulator of *miR-216a*. Strategies that inhibit *miR-216a* is a potential strategy for overcoming the cisplatin resistance in ovarian cancer.

## Introduction

Ovarian cancer is the fifth most common cancer amongst women in the United States. According to the estimation by American Cancer Society, approximately 22240 women will be newly diagnosed of ovarian cancer and approximately 14070 women will die from ovarian cancer in 2018. Despite progresses made in ovarian cancer therapy, the survival of patients with advanced ovarian cancer still remains dismal, due to the lack of effective treatment strategies to impede ovarian cancer metastasis and recurrence. Cisplatin, the platinum-based treatment, has been used as the first-line chemotherapy drug for ovarian cancer since more than 30 years ago [1,2]. However, little improvement has been made to the survival of ovarian cancer by cisplatin treatment, and metastatic and recurrent ovarian cancer frequently develop resistance, which primarily accounts for the poor clinical outcome of this treatment. It is imperative to develop novel methods to attenuate or reverse the cisplatin resistance in ovarian cancer.

Recent studies have implicated a plethora of factors involved in the ovarian cancer cisplatin resistance [3,4], including BRCA1 and BRCA2 mutations [5]. These discoveries, whereas, have yet to be translated into clinical practices. Human genome research unveiled a surprisingly high proportion of non-coding genes that play an indispensable role in many physiological processes. miRNAs (miRs) are a class of non-coding RNAs with the length of approximately 22 nts. Emerging evidences indicate that miRNAs are important regulators of cancer. Dysregulated miRNA expression substantially contribute to tumorigenesis and are associated with the poor clinical outcome of patients. A couple of miRNAs, such as *miR-214, miR-130a-3p*, and *miR-25-3p* have been identified as biomarkers of cancer cisplatin resistance [6–9]. These findings qualify miRNAs as a powerful toolkit for prevention, early detection, and therapy of cancers.

In ovarian cancer, the essential role of a number of miRNAs have been reported [10,11]. Altered expression of miRNA has been recognized as an important constituent of ovarian cancer [12,13]. PTEN is a putative tumor suppressor that regulates the oncogenic PI3K/Akt pathway [14]. Previous evidences have identified that *miR-214* is responsible for the enhanced cell survival and cisplatin resistance of ovarian cancer by targetting PTEN [6]. *miR-216a* is amongst the most investigated miRNAs in cancer [15–18]. It has been found that *miR-216a* is also an important mediator of PTEN [19].

Herein, we strive to clarify the correlation between *miR-216a* expression and malignant ovarian cancer and explore the mechanism of *miR-216a* in regulating cisplatin resistance in ovarian cancer. The effects of *miR-216a* up-regulation or inhibition on the phenotypical changes in two commonly used ovarian cancer cell lines, SKOV3 and OVCA433, were investigated. We are particularly interested in the downstream effector and upstream regulator of *miR-216a*. The results reported in the present study could provide clear evidence on the functional role of *miR-216a* and potentially provide an opportunity for the diagnosis, treatment of cisplatin-resistant ovarian cancers.

## Materials and methods

### Cell culture

SKOV3 and OVCA433 were acquired from American Type Culture Collection (ATCC, Rockville, MD, U.S.A.). Cells were cultured in RPMI-1640 medium supplemented with 10% FBS, in an incubator maintained at 37°C and 5% CO_2_. The cisplatin resistant SKOV3 CR cells were acquired by maintaining SKOV3 cells in the presence of cisplatin over a 10-month period. The cisplatin resistance sustained when SKOV3 CR cells were grown in the absence of cisplatin for 30 passages.

### Transfection of RNAs and plasmids

The miRNAs were purchased from GenePharma (Shanghai, China). *MiR-216a* mimics and *miR-216a* inhibitors (*miR-216a* siRNA), were transfected into ovarian cancer cells to induce *miR-216a* up-regulation and down-regulation, respectively. The PTEN plasmid was cloned into pcDNA3.1 plasmid and transfected into the cells using Lipofectamine 2000 (Invitrogen, Carlsbad, CA, U.S.A.) according to manufacturer’s protocols.

### Proliferation and soft agar colony formation assay

MTT assay was used to assess proliferation of cells. In brief, 2000 cells were seeded into 96-well plates at 24 h after treatment, the proliferation was monitored for 5 days. On each day, MTT reagent was added to a well and incubated for 2 h. Then, medium of the well was removed and 100 µl of DMSO was added and the absorbance was measured at 562 nm. Soft agar colony formation assay was performed according to a previously published protocol [20].

### qPCR assay

RNA was extracted from cells using the miRNeasy Mini Kit (Qiagen, Valencia, CA, U.S.A.) according to manufacturer’s recommendations. After purification, RNA was transcribed into cDNA using the High-Capacity cDNA Kit (Applied Biosystems, Waltham, MA, U.S.A.). Real-time PCR was then performed using the SYBR Green Master Mix (Applied Biosystems, U.S.A.). The primers used for qPCR include: PTEN sense 5′-TTGGCGGTGTCATAATGTCT-3′, antisense 5′-GCAGAAAGACTTGAAGGCGTA-3′; STAT3 sense 5′-TAGCAGGATGGCCCAATGGAATCA-3′, antisense 5′-AGCTGTCACTGTAGAGCTGATGGA-3′; GAPDH sense 5′-GAGTCAACGGATTTGGTCGT-3′, antisense 5′-TTGATTTTGGAGGGATCTCG-3′. The expression of GAPDH was used as a control. Quantitation of mRNA levels was performed using the 2^−ΔΔ*C*^_T_ method.

### Western blot

Western blot analysis was used to analyze PTEN expression. Proteins in SKOV3 cells were first extracted after cells’ lysis. Protein lysates of 20 µg were then used for SDS/PAGE, followed by transferring on to PVDF membranes. Rabbit anti-PTEN primary antibody was purchased from Abcam. The HRP–conjugated anti-rabbit secondary antibody was purchased from Abcam. PVDF membranes were blocked with 1% BSA for 1 h at room temperature. The anti-PTEN primary antibody diluted at 1:1000 in PBS-T (Tween 20, 0.1% v/v) was then added to the membrane and incubated at RT for 1 h with gentle shaking. After extensive washing with PBS-T, HRP–conjugated anti-rabbit antibody (1:20000 dilution) was added to the membrane and incubated at RT for another hour.

### Bioinformatics analysis and luciferase assay

TargetScan and rVista 2.0 were used for bioinformatics analysis. The prediction of effective miRNA target sites in mammalian mRNAs was performed using TargetScan using a previously published protocol [21]. The evolutionary analysis of transcription factor binding sites was performed using rVista according to previously published protocols [22]. Luciferase assay was used to confirm the interaction between two miRNAs and 3′-UTR of genes as previously described [16].

### Statistical analysis

SPSS was used for statistical analysis. Data were represented as mean ± S.D. Student’s *t* test was used to compare differences between two groups, and two-way ANOVA was used for comparison amongst three groups or between two groups with two factors. *P*-values of less than 0.05 were considered statistically significant.

## Results

### *MiR-216a* promotes ovarian cancer cells proliferation

**Figure 1:**
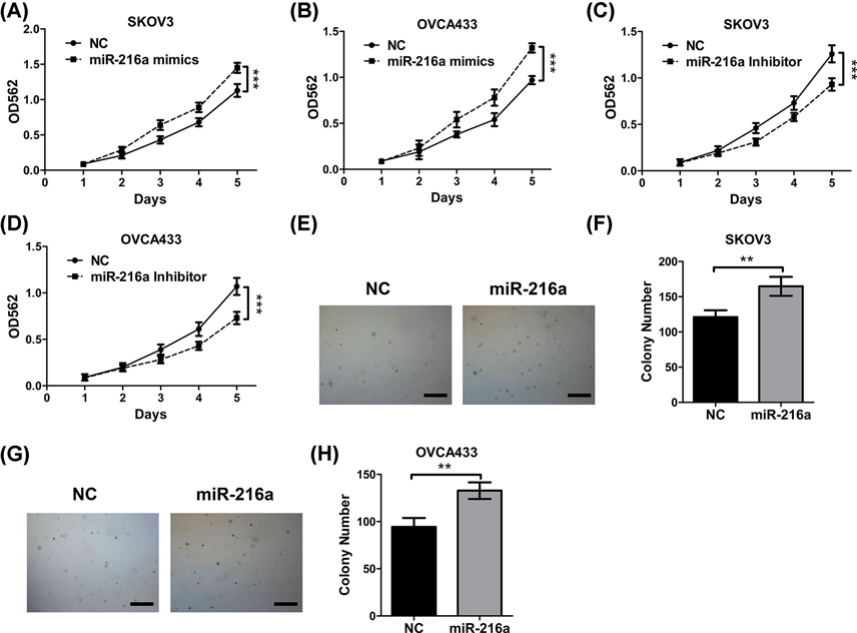
*MiR-216a* promotes ovarian cancer cells proliferation (**A**) The proliferation of SKOV3 cells transfected with *miR-216a* mimics was determined by MTT assay. (**B**) The proliferation of OVCA433 cells transfected with *miR-216a* mimics was determined by MTT assay. (**C**) The proliferation of SKOV3 cells transfected with *miR-216a* inhibitor was determined by MTT assay. (**D**) The proliferation of OVCA433 cells transfected with *miR-216a* inhibitor was determined by MTT assay. (**E**) The proliferation of SKOV3 cells transfected with *miR-216a* mimics was determined by soft agar colony formation assay. Scale bars: 500 μm. (**F**) The statistical results of soft agar colony formation assay. (**G**) The proliferation of OVCA433 cells transfected with *miR-216a* mimics was determined by soft agar colony formation assay. Scale bars: 500 μm. (**H**) The statistical results of soft agar colony formation assay. Data are shown as mean ± S.D. **P<0.01; ***P<0.001; (Student’s *t*test in (F,H) and others ANOVA test).

To clarify the role of *miR-216a* in ovarian cancer, *miR-216a* mimics or inhibitors were transfected into the SKOV3 and OVCA433 ovarian cells, followed by monitoring proliferation by MTT assay. As shown in Figure 1A–D, *miR-216a* mimics promoted the proliferation of SKOV3 (Figure 1A) and OVCA433 (Figure 1B), while *miR-216a* inhibitor attenuated proliferation of SKOV3 (Figure 1C) and OVAC433 (Figure 1D). This unveiled the tumor-promoting role of *miR-216a*. As another verification, colony formation assay, as shown in Figure 1E–H, demonstrated that *miR-216a* increased the number of colonies formed by SKOV3 (Figure 1E,F) and OVCA433 (Figure 1G,H) cells in soft agar. These data confirmed that *miR-216a* adopts a tumor-promoting role in ovarian cancer and the cancer cell proliferation is enhanced by *miR-216a*.

### *miR-216a* increases cisplatin resistance in ovarian cancer cells

**Figure 2:**
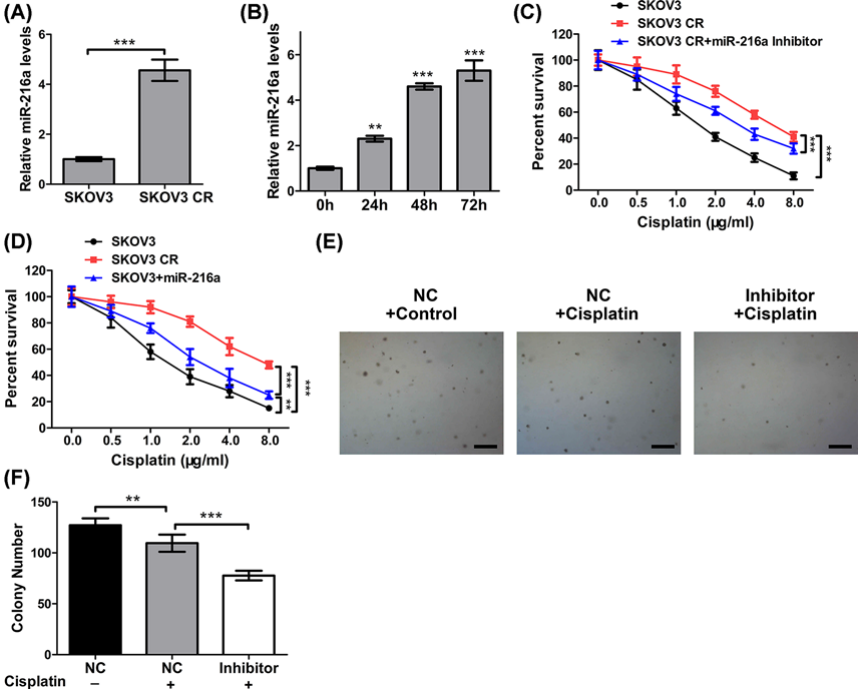
*MiR-216a* increases cisplatin resistance in ovarian cancer cells (**A**) The expression levels of *miR-216a* in SKOV3 and cisplatin-resistant SKOV3 CR cells was determined by real-time PCR. U6 was used as an input control. (**B**) The expression levels of *miR-216a* in SKOV3 cells treated with cisplatin (4 μg/ml) for different times was determined by real-time PCR. U6 was used as an input control. (**C**) Cell viability of SKOV3, SKOV3 CR cells transfected with or without *miR-216a* inhibitor treated with different concentrations of cisplatin was determined by MTT assay. (**D**) Cell viability of SKOV3 CR, SKOV3 cells transfected with or without *miR-216a* mimics treated with different concentrations of cisplatin was determined by MTT assay. (**E**) The proliferation of OVCA433 CR cells transfected with or without *miR-216a* inhibitor and treated with cisplatin (4 μg/ml) was determined by soft agar colony formation assay. Scale bars: 500 μm. (**F**) The statistical results of soft agar colony formation assay. Data are shown as mean ± S.D. **P<0.01; ***P<0.001; (Student’s *t*test in (F,H) and others ANOVA test).

We next examined the correlation between *miR-216a* and cisplatin resistance. Here we compared the miR-216a level in SKOV3 cells and that in cisplatin-resistant SKOV3 cells (SKOV3 CR). As shown in Figure 2A, *miR-216a* was markedly up-regulated in SKOV3 CR. Further, SKOV3 cells were treated with cisplatin (4 μg/ml) for varied duration (0–72 h), and consequently, an increasing *miR-216a* levels were seen with treatment duration (Figure 2B). Therefore, *miR-216a* up-regulation is also closely associated with cisplatin resistance. In-line with this, transfecting miR-216a inhibitor sensitized SKOV3 cells to cisplatin, as evidenced by lower survival with increasing cisplatin concentration (Figure 2C). Conversely, up-regulation of *miR-216a* through transfecting *miR-216a* mimics increased the resistance of SKOV3 to cisplatin (Figure 2D). Moreover, colony formation assay was performed for SKOV3, SKOV3 CR, and SKOV3 CR transfected with *miR-216a* inhibitor. As shown in Figure 2E,F, *miR-216a* inhibitor dramatically reduced the number of colonies. Consequently, inhibition of *miR-216a* was effective in attenuating cisplatin resistance.

### PTEN is a direct target of *miR-216a*

**Figure 3:**
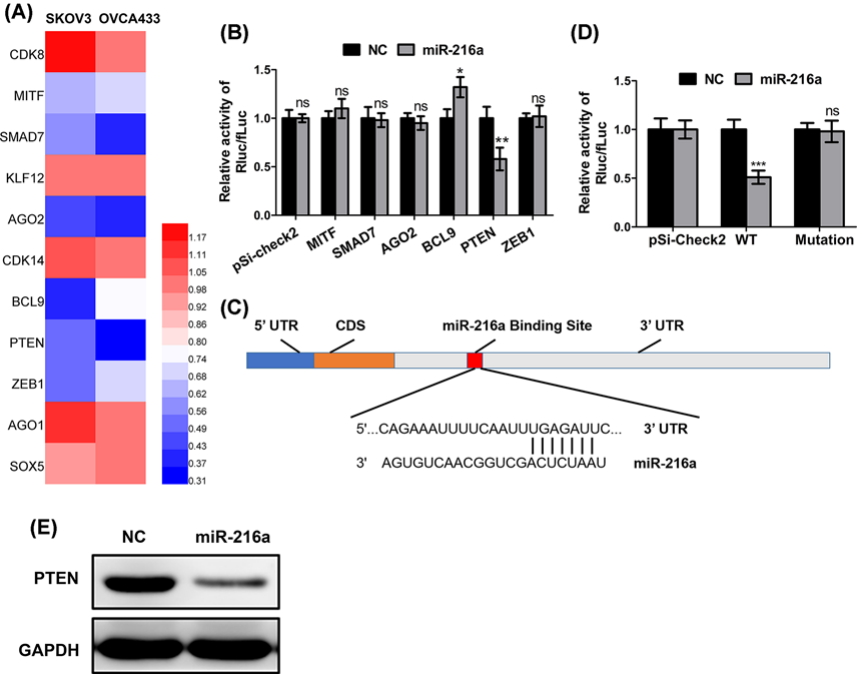
PTEN is a direct target of *miR-216a* (**A**) Heatmap representing the mRNA expression profile of a panel of cancer-related genes regulated by *miR-216a* in SKOV3 and OVCA433 cell lines determined using real-time PCR. (**B**) Luciferase reporter activities driven by the 3′-UTRs of potential *miR-216a* targets were examined in SKOV3 cells transfected with *miR-216a* mimics or negative control (NC). (**C**) Schematic diagram of *miR-216a* binding site on the 3′-UTR of *PTEN* mRNA. (**D**) Luciferase reporter activities driven by wild-type or mutant PTEN 3′-UTRs were examined in SKOV3 cells transfected with *miR-216a* mimics or NC. (**E**) Immunoblot analysis of PTEN protein level in SKOV3 cells transfected with *miR-216a* mimics. Data are shown as mean ± S.D. *P<0.05; **P<0.01; ***P<0.001; Abbreviation: ns, not significant (Student’s *t* test).

To unravel the factors involved in the cancer-regulatory role of *miR-216a*, we used TargetScan to predict targets of *miR-216a* and acquired 372 potential targets. We then performed a qPCR analysis of a panel of cancer-related genes in SKOV3 and OVCA433 cells transfected with non-coding miRNA or *miR-216a*. Figure 3A is a heatmap that represents the mRNA expression profile of eleven putative oncogenes, amongst which six showed down-regulation. Further, we cloned the 3′-UTR of those six genes to psi-check2 plasmid to perform dual-luciferase reporter assay. As shown in Figure 3B, we showed that only PTEN was significantly down-regulated by *miR-216a* mimic transfection (*P*<0.01). TargetScan study further identified the binding sites of *miR-216a* on PTEN (Figure 3C). To corroborate the binding site of *miR-216a* on PTEN, we mutated the binding site on the 3′-UTR of PTEN and consequently abrogated the down-regulation of PTEN by *miR-216a* (Figure 3D). In addition, Western blot analysis was performed, showing that PTEN was down-regulated by *miR-216a* mimics transfection (Figure 3E).

### PTEN is the functional downstream effector of *miR-216a*

**Figure 4:**
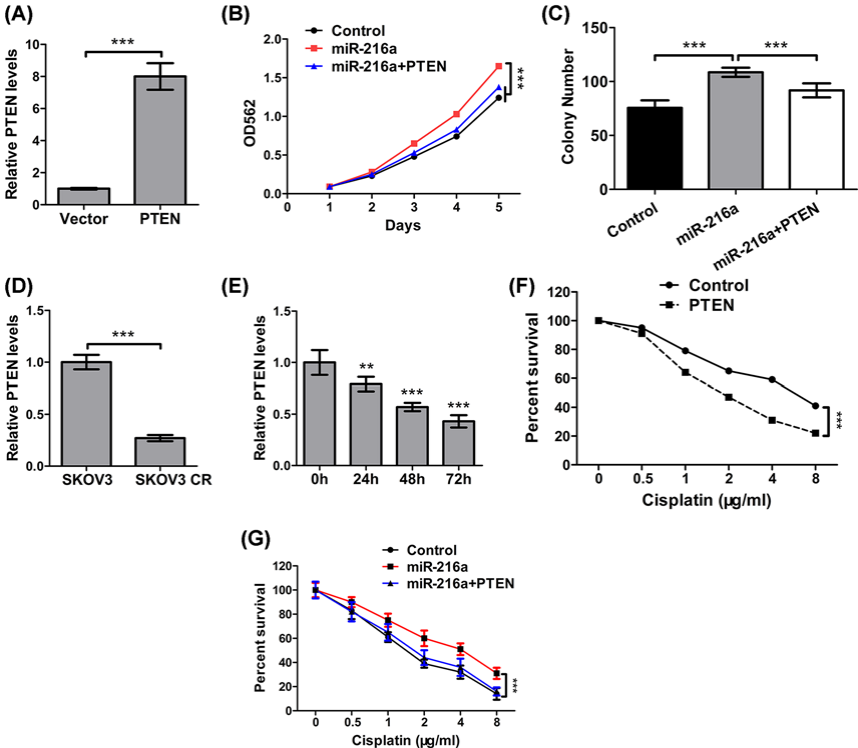
PTEN is the functional downstream effector of *miR-216a* (**A**) The mRNA expression of PTEN in SKOV3 cells transfected with PTEN overexpression plasmid was determined by qPCR. (**B**) Cell viability of SKOV3 cells transfected with *miR-216a* or *miR-216a* and PTEN was determined by MTT assay. (**C**) Cell viability of SKOV3 cells transfected with *miR-216a* or *miR-216a* and PTEN was determined by soft agar assay. (**D**) The mRNA levels of PTEN in SKOV3 and SKOV3 CR cells were determined by qPCR. (**E**) The mRNA levels of PTEN in SKOV3 cells treated with cisplatin (4 μg/ml) for different time were determined by qPCR. (**F**) Cell viability of SKOV3 cells transfected with or without PTEN and treated with different concentration of cisplatin was determined by MTT assay. (**G**) Cell viability of SKOV3 cells transfected with *miR-216a* and/or PTEN and treated with different concentration of cisplatin was determined by MTT assay. **P<0.01; ***P<0.001.

We further explored whether *miR-216a* promotes ovarian cancer proliferation and metastasis through PTEN. To this end, we induced PTEN overexpression in SKOV3 cells. qPCR analysis was used to confirm the overexpression of PTEN (Figure 4A). After transfection with *miR-216a* and/or PTEN for 24 h, the cell proliferation was determined by MTT and soft agar assay.

Consistent with the role of *miR-216a* in down-regulating PTEN, overexpression of PTEN in cells transfected with *miR-216a* resulted in retarded cell proliferation (Figure 4B) and colony formation (Figure 4C). More importantly, we showed that cisplatin-resistant cells were characterized by dramatic down-regulation of PTEN (*P*<0.001) (Figure 4D), and PTEN down-regulation became increasingly prominent with the increasing duration (1–3 days) of cisplatin treatment (Figure 4E). At day 3, the level of PTEN was less than 50% of control cells (*P*<0.001). These data implied that PTEN down-regulation is correlated with cisplatin-resistance. As another verification, we overexpressed PTEN in SKOV3 cells and observed an increased cell survival with increasing dose of cisplatin (Figure 4F). Furthermore, *miR-216a* decreased the sensitivity to cisplatin in SKOV3 cells, while overexpressing PTEN reversed this effect (Figure 4G). Taken together, PTEN down-regulation, induced by *miR-216a* up-regulation, is indispensable for cisplatin resistance of ovarian cancer cells.

### *MiR-216a* is regulated by STAT3

**Figure 5:**
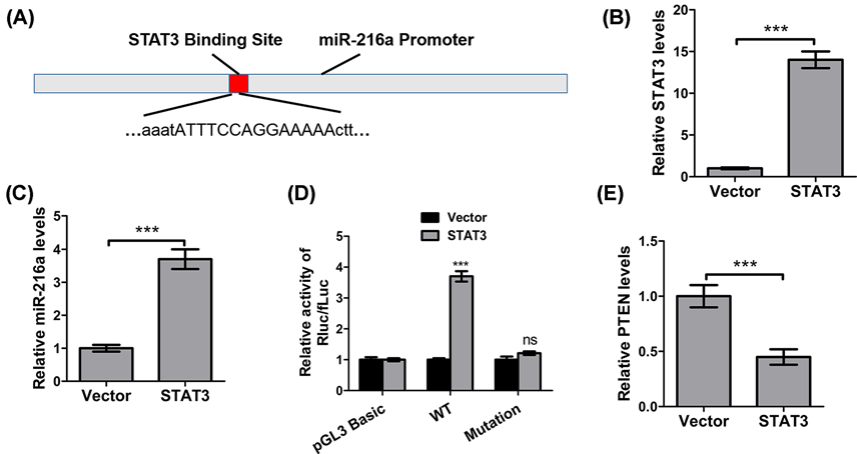
*miR-216a* is regulated by STAT3 (**A**) Schematic diagram of STAT3 binding sites on the promoter of *miR-216a*. (**B**) The expression of STAT3 in SKOV3 cells transfected with vector or STAT3 expression plasmid. (**C**) The expression of *miR-216a* in SKOV3 cells transfected with vector or STAT3 overexpression plasmid. (**D**) Luciferase assays for *miR-216a* promoter (wild-type or mutant) activity. The promoter constructs were co-transfected with PTEN expressing plasmid. (**E**) The expression of PTEN in SKOV3 cells transfected with vector or STAT3 expression plasmid. ***P<0.001. ns, not significant.

We next strived to elucidate what contributes to the up-regulation of *miR-216a* in ovarian cancer. Using rVista 2.0 for binding region prediction, we uncovered that *miR-216a* promoter contains a binding region for STAT3 (Figure 5A). To validate the interaction between STAT3 and *miR-216a*, we overexpressed STAT3 in SKOV3 cells, which was confirmed by qPCR (Figure 5B), followed by evaluating *miR-216a* levels in STAT3 overexpressing cells. As shown in Figure 5C, we also observed a marked up-regulation of *miR-216a* in cells with high STAT3 expression. Dual-luciferase reporter assay verified that while STAT3 overexpression induced substantial up-regulation luciferase activity (Figure 5D), mutation of *miR-216a* promoter region abrogated this effect. Consistent with the aforementioned antagonizing effects between *miR-216a* and PTEN, STAT3 overexpression also significantly reduced PTEN levels significantly (*P*<0.001) (Figure 5E). These data clearly suggested that STAT3 governs the overexpression of *miR-216a*, which consequently reduces PTEN expression.

## Discussion

Here we demonstrate the important role of *miR-216a* in cisplatin resistance of ovarian cancer. Thus far, *miR-216a* has been reported as both a tumor inducer and a tumor suppressor [16,17,23] and the role of *miR-216a* in ovarian cancer still remains elusive. Additionally, the link between *miR-216a* and cancer resistance has rarely been established. Previously, Xia et al. [16] showed that *miR-216a/217* induces epithelial-to-mesenchymal transition (EMT) through targetting PTEN, thereby promoting drug resistance and recurrence of liver cancer. The up-regulation of *miR-216a*, which can be detected by measuring *miR-216a* levels in plasma, has also demonstrated potential as a diagnostic marker in pancreatic cancer [24]. However, to our knowledge, our study represents the first evidence that *miR-216a* increases cisplatin resistance in ovarian cancer. What has not been demonstrated here is whether *miR-216a* possesses diagnostic value in ovarian cancer. In this context, the measurement of plasma *miR-216a* levels can also be adopted to study the correlation of *miR-216a* up-regulation and clinical outcomes of ovarian cancer patients. No effective screening method is available to detect early-stage ovarian cancer with high sensitivity and specificity. The close alliance between *miR-216a* up-regulation and cisplatin may allow to identify the patients who do not respond to cisplatin therapy, consequently facilitating timely and efficient treatment planning for this highly lethal disease.

Our study provides a new avenue for overcoming cisplatin resistance to improve the clinical outcomes of patient with ovarian cancers at advanced stages. Currently, the overall 5-year survival rates of patients diagnosed at stages III and IV of this disease are 32 and 18%, respectively [2]. A large population of ovarian cancer patients, who initially respond to standard chemotherapeutic regimens, inevitably relapse with recurrence, metastasis, and drug resistance. Because of this, conventional cytotoxic chemotherapy with cisplatin, even in combination with paclitaxel, have not had a significant impact on overall survival of ovarian cancer in last several decades. Our efforts in elucidating the mechanisms of cisplatin resistance and devising approaches to overcome the resistance are juxtaposed with numerous studies that focus on miRNAs as promising cancer targets [6,25–27]. The inhibition of these miRNAs offers a precise and potent tool to throttle oncogenic pathways that drive cisplatin resistance. Here we utilized siRNA specific to *miR-216a* to inhibit *miR-216a* expression, which is a clinically applicable approach. Fueled by recent advances in moving siRNA therapeutics from bench to clinics [28], possible therapeutic strategy can also be developed by suppressing *miR-216a* expression. It should be noted that cisplatin resistance may also be ascribed to other mechanisms, such as reduced accumulation of the drug [29], increased levels of metallothionein [30] and gluotathione [31], as well as enhanced DNA repair [32]. Gene predisposition, such as BRCA1 and BRCA2 mutation, also contributes significantly to ovarian cancer. Successful treatment of ovarian cancer may necessitate a comprehensive strategy based on these resistance mechanisms, in combination to cisplatin therapy.

We identified PTEN as the downstream effector of *miR-216a*. We also analyzed a panel of putative cancer-related genes, including *CDK8, MITF, SMAD7, KLF12, AG02, CDK14*, and *BCL-9* [33–39], but only PTEN expression was shown to be significantly altered by *miR-216a* overexpression. The loss of PTEN has been implicated as an important mediator of EMT [40], which enhances cancer cells proliferation, migration, and metastasis, thereby leading to poor survival rates of patients. Due to the regulatory role of PTEN in PIK3/Akt pathway, and their critical role in cell apoptosis, it is quite reasonable that resultant inhibition of PTEN by *miR-216a* led to reduced apoptosis by cisplatin [41]. This observation is consistent with the notion that the suppressed apoptosis is a major contributing factor to cisplatin resistance in ovarian cancers [42].

To elucidate the underlying mechanism of *miR-216a* in ovarian cancer regulation, we also demonstrated that STAT3, a putative oncogene in ovarian cancer [43], is a regulator of *miR-216a*. Previous studies have suggested the interaction between *miR-216a* and JAK2/STAT pathway [17,18]. However, in these studies STAT3 is a target of *miR-216a*. STAT3 induces cellular transformation and promotes tumorigenesis by regulating a wide array of oncogenes, including c-Myc, cyclinD1 etc. [44]. In the present study, we showed that STAT3 promotes *miR-216a* by binding to the promoter region of *miR-216a*. These data are in accordance with previous evidences that STAT3 regulates the expression of a number of miRNAs [45]. By regulating *miR-216a*, PTEN was suppressed by STAT3 overexpression. In-line with this, the interaction of PTEN and STAT3 has been demonstrated previously and their levels of expression were shown to be negatively correlated [45,46]. Given the significant roles of STAT3 and PTEN, and the close interaction of *miR-216a* with both of them, *miR-216a* is an important molecule in cancer and further investigations are warranted to further demonstrated the clinical utility of *miR-216a* in ovarian cancer.

## Conclusion

Here we show that *miR-216a* is an inducer of cisplatin resistance in ovarian cancer. Inhibition of *miR-216a* attenuated the cisplatin resistance. Using bioinformatics approach and further validation with qPCR and Western blot, we show that PTEN is an effector and STAT3 is a regulator of *miR-216a*. By elucidating the role and mechanism of *miR-216a* in ovarian cancer, our study provides an opportunity to overcome cisplatin resistance in ovarian cancer.

## Abbreviation

EMT: epithelial-to-mesenchymal transition
PTEN: phosphatase and tensin homolog
